# The Outcomes of Mini-Plate Fixation for Unstable Wagstaffe Tubercle Fracture, an Indirect Syndesmosis Injury in Rotational Ankle Fracture

**DOI:** 10.3390/jcm13061605

**Published:** 2024-03-11

**Authors:** Byung-Ryul Lee, Ki-Jin Jung, Eui-Dong Yeo, Sung-Hun Won, Yong-Cheol Hong, Chang-Hwa Hong, Chang-Hyun Kim, Ho-Sung Kim, Jae-Young Ji, Je-Yeon Byeon, Dhong-Won Lee, Woo-Jong Kim

**Affiliations:** 1 Department of Orthopaedic Surgery, Soonchunhyang University Cheonan Hospital, 31, Suncheonhyang 6-gil, Dongam-gu, Cheonan 31151, Republic of Korea; 129027@schmc.ac.kr (B.-R.L.); c89546@schmc.ac.kr (K.-J.J.); 121806@schmc.ac.kr (Y.-C.H.); chhong@schmc.ac.kr (C.-H.H.); s99942@schmc.ac.kr (C.-H.K.); 2 Department of Orthopaedic Surgery, Veterans Health Service Medical Center, Seoul 05368, Republic of Korea; angel_doctor@naver.com; 3 Department of Orthopaedic Surgery, Soonchunhyang University Seoul Hospital, 59, Daesagwan-ro, Yongsan-gu, Seoul 04401, Republic of Korea; orthowon@schmc.ac.kr; 4 Department of Orthopaedic Surgery, Soonchunhyang University Bucheon Hospital, 170, Jomaru-ro, Bucheon 14584, Republic of Korea; nine4141@naver.com; 5 Department of Anesthesiology and Pain Medicine, Soonchunhyang University Hospital Cheonan, 31, Suncheonhyang 6-gil, Dongam-gu, Cheonan 31151, Republic of Korea; phmjjy@naver.com; 6 Department of Plastic Surgery, Soonchunhyang University Hospital Cheonan, 31, Suncheonhyang 6-gil, Dongam-gu, Cheonan 31151, Republic of Korea; wpdusqus@gmail.com; 7 Department of Orthopaedic Surgery, Konkuk University Medical Center, 120-1, Neungdong-ro, Gwangjin-gu, Seoul 05030, Republic of Korea; bestal@naver.com

**Keywords:** ankle fracture, syndesmosis injury, Wagstaffe tubercle, avulsion fracture, mini-plate fixation

## Abstract

**Background:** Wagstaffe fracture constitutes an indirect injury to the AITFL and can precipitate syndesmotic instability. The prevailing fixation methods often involve the use of mini-screws or K-wires, with absorbable suture repair reserved for cases with small or comminuted fragments exhibiting instability. In this study, we devised a mini-plate fixation method capable of securing the fracture fragment irrespective of its size or condition. **Methods:** A retrospective chart review was conducted on patients who underwent surgery for ankle fractures between May 2022 and October 2023. The surgical technique involved direct fixation of the Wagstaffe fracture using mini-plate fixation. Radiologic evaluation was performed using postoperative CT images, and clinical outcomes were assessed using the OMAS and VAS. **Results:** Fourteen patients with an average age of 62.5 years were included. Most fractures were associated with the supination-external rotation type. The average preoperative OMAS significantly improved from 5.95 to 83.57 postoperatively. The average VAS score decreased from 7.95 preoperatively to 0.19 postoperatively. **Conclusions:** The mini-plate technique for Wagstaffe fractures exhibited dependable fixation strength, effective fracture reduction, a minimal complication rate, and judicious surgical procedure duration.

## 1. Introduction

A syndesmosis is a group of bones and ligaments that connects the fibula and tibia at the ankle. It includes the anterior inferior tibiofibular ligament (AITFL), posterior inferior tibiofibular ligament (PITFL), and the interosseus membrane (IOL). Syndesmosis injuries, which account for approximately 10% of rotational ankle fractures, require surgery in over 20% of cases, as reported in various studies [1,2,3]. Syndesmosis plays a crucial role in maintaining the lattice structure of the ankle and restoring congruity, which is important for positive long-term outcomes and the prevention of posttraumatic osteoarthritis in ankle fractures. Surgery becomes necessary in cases with unstable syndesmosis, which can occur across various ankle fracture patterns, including trimalleolar, bimalleolar, and unstable lateral malleolar fractures. Regardless of the fracture type, it is essential to assess all surgical ankle fractures for associated syndesmosis instability, which can manifest independently of fractures [4,5,6].

The anterior inferior tibiofibular ligament (AITFL) is the primary contributor to syndesmosis stability in the ankle [7]. Due to its anatomical position, the AITFL can be injured during the external rotation mechanism of the ankle, as described by Lauge-Hansen. This injury can either be to the ligament itself or a bony avulsion [8].

The fracture of the Wagstaffe tubercle, where the AITFL attaches, commonly known as a “Wagstaffe fracture,” constitutes an indirect injury to the AITFL and may lead to syndesmosis instability [9,10,11]. Haraguchi et al. reported that 65% of avulsion fractures of the lateral ankle ligaments treated conservatively achieved osseous union, while 35% of the patients experienced nonunion, comprising over one-third of the total patient population. This nonunion can become symptomatic in some cases, causing chronic ankle pain and instability, potentially hindering athletic activities or strenuous work [12].

Recent studies have reported positive outcomes of the direct fixation of Wagstaffe fractures [10,11,13,14]. This technique emerged as a reliable alternative for managing Wagstaffe fractures and the associated syndesmosis instability, leading to improved long-term functional outcomes compared to indirect trans-syndesmotic screw fixation. The commonly employed fixation methods include mini-screws or K-wires, with absorbable suture repair applied in cases of small or comminuted and unstable fragments [10,11,13]. However, the applicability of these fixation methods is limited by the fragment’s size and condition.

In response to these findings, we developed a mini-plate fixation method capable of securing the fracture fragment regardless of its size or state, thereby enhancing fixation strength. In this study, we present our surgical results using this method to treat Wagstaffe fractures.

## 2. Materials and Methods

### 2.1. Patient Selection

This study, approved by the Institutional Review Board of Soonchunhyang University Cheonan Hospital (approval no. 2023-10-037), was a retrospective chart review of patients who underwent ankle fracture surgery between May 2022 and October 2023. The exclusion criteria were designed to facilitate the selection of patients for comparison with healthy counterparts and to ensure the examination of complications in individuals without concurrent soft tissue injuries. These criteria included the following: (1) age < 18 years, (2) a follow-up period shorter than 1 year, (3) an open ankle fracture, (4) an ipsilateral fracture extending to the tibial plafond, (5) a history of ankle fracture or syndesmotic injury in either ankle, (6) a history of contralateral ankle surgery, and (7) a history of arthritis in either ankle. This study investigated the demographic features of patients with avulsion fractures by including individuals of all types. Prior to participation, informed consent was obtained from each patient or their appropriate family member. The analysis included factors such as age, sex, and the mechanism of injury. Fractures were diagnosed using various imaging techniques, including X-rays and 3D computed tomography (CT) scans. The Lauge-Hansen classification method was used to classify the ankle fractures, while AITFL avulsion fractures were categorized using the modified Wagstaffe classification method.

### 2.2. Clinical Evaluations

The patients were followed-up with radiologic evaluations at 2 weeks, 4 weeks, 6 weeks, 3 months, 6 months, and 12 months after the surgery. The Olerud Molander Ankle Score (OMAS) was used to assess overall functionality and the subjective satisfaction of patients recovering from ankle injuries. Additionally, a visual analog scale (VAS) was used to measure pain levels [15].

### 2.3. Radiological Evaluations

**Figure 1:**
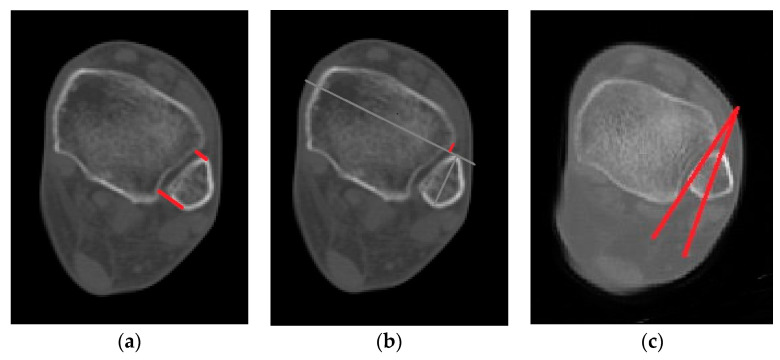
Radiologic measurements. (Red line) (**a**) Direct anterior difference and direct posterior difference. (**b**) Fibular translation. (**c**) Fibular rotation (Red line).

This study evaluated syndesmosis reduction by analyzing postoperative axial CT images captured 1 cm proximal to the tibial plafond (Table 1 and Figure 1) [16,17,18,19]. Four radiographic measurements were chosen and assessed with a PACS image viewer software (Dejaview2 version 1.0, Dongwun Information Technology, Republic of Korea). Two independent observers, blinded to patients’ clinical outcomes and current complaints, objectively evaluated the measurements. The process was repeated after a 6-week interval to ensure reliability.

### 2.4. Surgery and Rehabilitation

**Figure 2:**
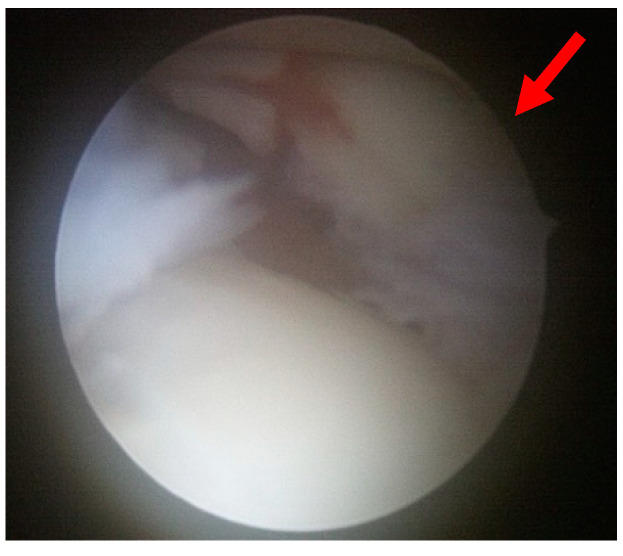
An avulsed Wagstaffe tubercle fracture (red arrow) is observed on arthroscopy.

All procedures were performed by a single surgeon (W.J.K.). Patients were administered either general or spinal anesthesia or a lower extremity nerve block and were positioned supine during the surgery. The surgical area was cleaned and draped in a sterile manner, with a tourniquet inflated to ensure a clear view of the surgical field. The joint was irrigated through arthroscopy and was subsequently examined for any joint lesions or syndesmosis instability (Figure 2). Instability was defined as the tibiofibular joint space exceeding 2 mm upon probing or when the ankle was externally rotated.

The surgical process involved an anterolateral incision from the proximal part of the lateral malleolar fracture site to the Wagstaffe fracture site. Following the reduction in and fixation of the lateral malleolar fracture, the stability of the syndesmosis was re-evaluated.

**Figure 3:**
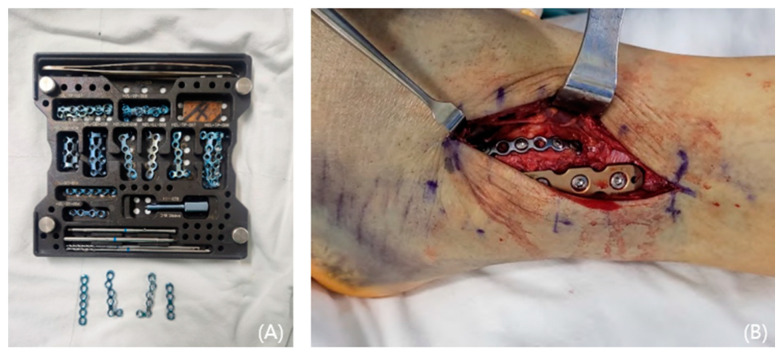
(**A**) A mini-plate set (Arix Hand System, Jeil Medical, Seoul, Republic of Korea). (**B**) The Wagstaffe fracture is fixed with a dorsal mini-plate.

**Figure 4:**
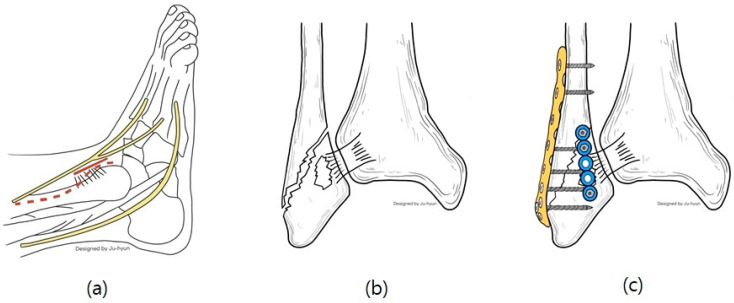
Surgical technique for fixing Wagstaffe tubercle fracture with a mini plate: (**a**) for a fibular fracture, a curved anterolateral approach (dotted line) was used to expose the Wagstaffe fragment at the insertion of the AITFL. If the fibula fracture did not need reduction, the Wagstaffe fracture site was exposed using only the solid line incision. (**b**) In addition to the fibula fracture, a Wagstaffe fragment fracture connected to the AITFL was observed from the front. (**c**) The fracture site was compressed and fixed with a mini-plate after reduction.

**Figure 5:**
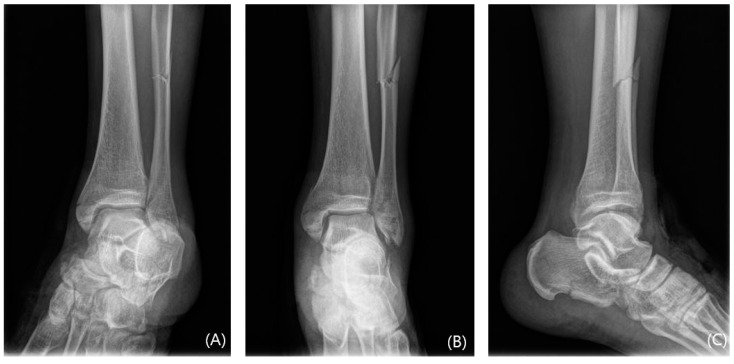
The preoperative mortise, anteroposterior, and lateral radiographs (**A**–**C**) of a 47-year-old female with Lauge-Hansen class PER IV ankle fracture. The AITFL fibular avulsion fracture is not visible on these plain radiographs.

**Figure 6:**
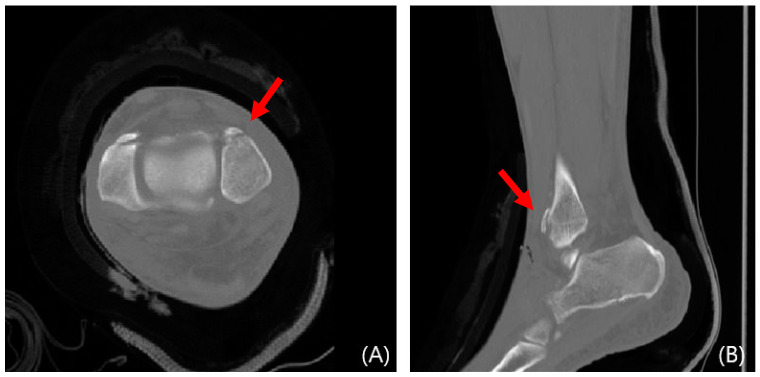
Preoperative CT images of a 47-year-old female with a Wagstaffe fracture (red arrow). Axial (**A**) and sagittal (**B**) views.

**Figure 7:**
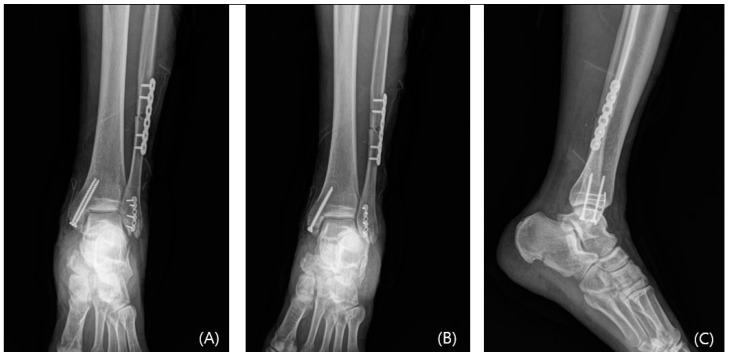
The postoperative mortise, anteroposterior, and lateral radiographs (**A**–**C**) showing mini-plate fixation for Wagstaffe tubercle fracture.

**Figure 8:**
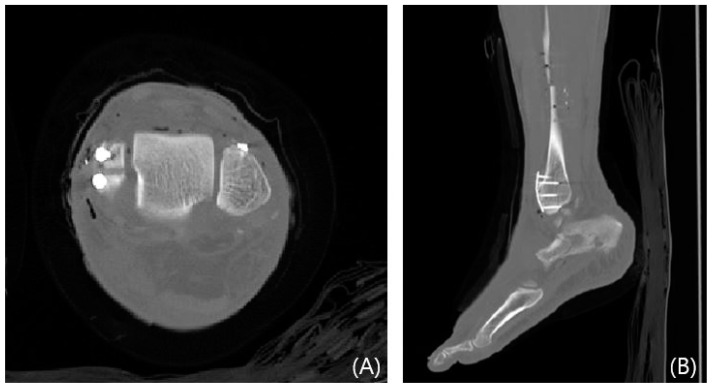
Postoperative CT images showing mini-plate fixation for a Wagstaffe tubercle fracture. Axial (**A**) and sagittal (**B**) views.

In cases of instability, a mini-plate (Arix Hand System, Jeil Medical, Seoul, Republic of Korea) is shaped to cover the Wagstaffe fracture and secured using 2.0 mm cortical screws and locking screws (Figure 3). For smaller fractures, screws were applied to the proximal and distal sections of the mini-plate at the fracture site. Screw fixation was performed at the fracture site when possible (Figure 4). Subsequently, tibiofibular joint stability was assessed through arthroscopy. Figure 5 and Figure 6 show the preoperative plain X-ray and CT images of a 47-year-old female with a Wagstaffe fracture. Figure 7 shows the postoperative plain X-ray image of a Wagstaffe fracture treated with open reduction and internal fixation using the described technique. Postoperative CT images were used to further confirm the reduction and fixation (Figure 8).

Following the surgery, the lower limb was immobilized using a cast or walking brace for the initial 4 weeks. Subsequently, joint range of motion exercises were initiated while the patient wore a walking brace, gradually transitioning from partial weight-bearing to full weight-bearing within 6 weeks postoperatively. The brace was removed 6 weeks postoperatively.

### 2.5. Statistical Analysis

An expert statistician conducted the statistical analysis using SPSS software version 29.0 (IBM Corp., Armonk, NY, USA). Continuous variables were presented as the mean ± standard deviation (SD). Student’s *t*-test was used to compare pre- and post-operative VAS and OMAS scores. Statistical significance was set at *p* < 0.05 for two-sided tests. Inter- and intra-observer reliability was evaluated for each assessment method. To assess inter- and intra-observer reliability for all methods involving continuous variables, intraclass correlation coefficients (ICCs) were calculated using a two-way mixed-effects model.

## 3. Results

A total of 17 patients with AITFL avulsion fracture and displaced Wagstaffe tubercle fracture were included in this study, including 7 males and 10 females, with an average age of 63.18 years (range: 44–80 years). The sample included 5 right-sided and 12 left-sided fractures. The patients were followed-up for an average of 16.18 months. The Lauge-Hansen classification type supination-external rotation accounted for the majority of the cases (*n* = 12/14; Table 2).

### 3.1. Clinical Outcomes

Both the OMAS and VAS scores demonstrated significant improvement following surgery. The average OMAS score increased substantially from 5.88 preoperatively (ranging from 0 to 35) to 83.53 postoperatively (ranging from 60 to 95) (*p* < 0.001). Similarly, the average VAS score, indicative of pain levels, significantly decreased from 8.11 preoperatively (ranging from 7 to 9) to 0.59 postoperatively (ranging from 0 to 2) (*p* < 0.001). None of the patients required reoperation due to complications. Table 3 presents a comprehensive overview of patient demographics and the clinical analysis, detailing information about the characteristics of the study population and clinical assessment findings.

### 3.2. Radiologic Outcomes

Table 4 presents the radiologic measurements obtained by the first and second observers for all evaluation methods, while Table 5 presents the agreement results. Comparisons between the normal (*n* = 17) and damaged (*n* = 17) sides using the evaluation method revealed a direct anterior difference of 4.57 mm (SD 1.25), a direct posterior difference of 8.42 mm (SD 1.18), a fibular translation of 1.99 mm (SD 1.13), and fibular rotation of 11.52° (SD 5.55) on the normal side, while the values on the damaged side were 4.46 mm (SD 1.03), 8.94 mm (SD 2.02), 1.67 mm (SD 0.67), and 13.35° (SD 6.41), respectively. Similar patterns were observed by the second observer, with a direct anterior difference of 4.49 mm (SD 1.12), a direct posterior difference of 8.32 mm (SD 1.14), a fibular translation of 1.88 mm (SD 1.06), and fibular rotation of 11.31° on the normal side. The values on the damaged side were 4.67 mm (SD 1.05), 9.12 mm (SD 1.84), 1.73 mm (SD 0.70), and 13.43° (SD 6.47), respectively.

There were no significant differences observed in the measured values between the normal and injured sides for each evaluation method. The intraobserver reliability for the four radiological measurements ranged from 0.779 to 0.948 and from 0.854 to 0.940 for the first and second observers, respectively. The interobserver reliability for the four radiological measurements ranged from 0.918 to 0.975 between the two observers.

### 3.3. Complications

Few complications were observed among the 17 patients. One patient reported discomfort in the surgical area while walking. This was thought to be due to the proximity of the small metal plate to the Chaput tubercle, causing joint irritation. The symptoms resolved once the metal plate was removed following bone union. Another patient had a minor local infection, which improved with antibiotic treatment.

## 4. Discussion

The distal syndesmotic articulation between the tibia and fibula comprises the following three major ligaments: AITFL, PITFL, and IOL [4]. The AITFL plays a pivotal role in preventing the lateral translation of the fibula, providing rotational stability that hinders approximately 24% of external rotation at the ankle joint [20]. Ogilvie et al. stated that the relative importance to the stability of the four component ligaments of the distal tibiofibular syndesmosis is as follows: AITFL at 35%, the interosseous ligament at 22%, superficial posterior inferior tibiofibular at 9%, and deep posterior inferior tibiofibular at 33% [7]. Therefore, precise diagnosis and suitable treatment have the potential to significantly influence the outcomes for the ankle joint.

In 1875, Wagstaffe documented instances of avulsed fragments in the distal fibula concomitant with ankle fractures [21]. Subsequently, in 1886, LeFort identified a vertical fracture in the anteromedial aspect of the fibula, specifically at the Wagstaffe tubercle, proposing its correlation with AITFL insertion [22]. The reported incidence of Wagstaffe ankle fractures varies between 1.5% and 25.8% [15]. Initially, the fracture was categorized into three types based on the Wagstaffe classification system. However, in 1979, Pankovich et al. reclassified it, expanding the classification to four types. Type I fractures involve a solitary fracture of the anterior fibular tubercle; type II fractures combine a fracture of the anterior fibular tubercle with a fibular fracture; type III fractures encompass a fibular fracture and a fracture of the anterior incisura of the tibia; and type IV fractures involve the anterior fibular tubercle, combined with a fibular fracture and a fracture of the anterior incisura of the tibia [9].

Wagstaffe type II and type III fractures generally have a similar incidence. In a study on nine cases of Wagstaffe fractures, Pankovich reported a high prevalence of type II fractures, accounting for eight cases [20]. In another study, Park et al. documented 13 cases of Wagstaffe type II fractures among a total of 94 cases [9]. Chung et al. conducted a study on 30 Wagstaffe fracture cases, where type II fractures occurred most frequently, accounting for 23 fractures (77%) [13]. Furthermore, the majority were concomitant with supination-external rotation-type fractures.

Wagstaffe fractures represent a specific fracture pattern involving the lower end of the fibula and AITFL avulsion, typically caused by the external rotation of the ankle joint and often associated with ankle joint dislocation [18]. Unlike typical lateral ankle sprains caused by inverted and plantar-flexed foot positions, syndesmosis injuries arise from different mechanisms. These often involve forceful external rotations, the eversion of the talus, or dorsiflexion, all of which widen the ankle joint and separate the fibula from the tibia. This persistent instability can increase shear stress and pressure patterns within the joint, potentially leading to cartilage degeneration over time [23]. The present study is in agreement with these trends, with type II fractures (supination-external rotation-type) being the most prevalent, accounting for 15 (88%) of cases. Meanwhile, type IV fractures involving both anterior tubercles of the distal tibia and distal fibula are uncommon and poorly understood [15].

In Weber B fractures, ankle diastasis was common, and Wagstaffe fractures were strongly associated with ankle diastasis. The proper reduction and rigid fixation of displaced lateral malleolar fractures are considered essential, highlighting the importance of addressing syndesmosis, particularly in Wagstaffe fractures. There is evidence suggesting that the correct positioning of the distal fibula into the tibial incisura is prognostically relevant in malleolar fractures [23]. This study emphasizes the need for careful examination during ankle surgeries to achieve proper reduction and fixation, thereby preventing postoperative complications, such as pain, instability, and arthrosis.

Treatment typically involves immobilization with a cast or splint, and in severe cases, surgical intervention might be necessary, depending on fracture severity and individual patient circumstances. Seeking prompt medical attention for a thorough evaluation and appropriate treatment is crucial in suspected Wagstaffe fractures or ankle injuries [9]. Despite ongoing discussions, there is a lack of consensus on the optimal surgical treatment for syndesmotic injuries. However, most authors agree that syndesmosis malreduction is associated with unfavorable clinical outcomes. Several studies have consistently demonstrated that neglecting these fractures could lead to suboptimal reduction in the ankle mortise, necessitating further corrective procedures [18,24]. In a previous study, the authors of this study reported reliable functional and radiological outcomes in 21 Chaput fracture patients (Wagstaffe type-III) who underwent fragment fixation using the tension band wire technique [25,26]. Previous research findings indicated that when indirect syndesmosis instability was present, direct fixation alone provided sufficient stability, eliminating the need for transfixation screws. Therefore, we hypothesized that direct fixation would also yield favorable outcomes in Wagstaffe fractures, leading to the development of the mini-plate fixation method.

Regarding Wagstaffe fractures, the current body of evidence lacks comprehensive support for an optimal treatment approach or anatomical validation of the fracture’s morphology and its correlation with instability. Fragment size appears to impact the fixation method for the avulsed fragment. This study proposes a novel AITFL avulsion fracture classification system, emphasizing the role of fragment size in fixation methods, with larger fragments (≥5 mm) significantly correlated with direct fixation [27]. Haraguchi et al. reported only a 65% rate of union for non-operated Chaput fractures [12]. Zhao et al. reported an 80% success rate with open reduction/internal fixation in a study of 15 adult patients with ankle fractures, including Tillaux–Chaput fractures, with most cases rated excellent or good on the AOFAS [28]. Bae et al. performed direct avulsion fracture fixation in cases of syndesmotic instability following malleolar fractures, achieving stability in 83.3% of cases, with 16.7% requiring additional syndesmosis screw fixation [11]. Chung et al. used K-wires, mini-screws, or absorbable suture materials in a study on 30 cases [13], including 28 Lauge-Hansen classification supination-external rotation type cases. The internal fixation involved mini-screws in 11 cases, K-wire in 10 cases, repair in 6 cases, and a combination of mini-screws and K-wires in 3 cases. Bony union was achieved in all cases. Meanwhile, Rammelt et al. used plates, screws, and suture anchors, resulting in excellent treatment outcomes [18]. They detected AITFL avulsion fractures through meticulous examination methods, including simple radiography, computed tomography, and surgical findings, followed by precise internal fixation to avert potential complications.

Gasparova et al. suggested that screw fixation is suitable for single-fragment fractures, while plate fixation is more effective for fractures with multiple fragments [29]. The use of screw fixation for AITFL avulsion fractures has been reported previously. However, the application of plate fixation, as in this study, remains under-reported. The mini-plate technique presents a viable option for achieving stability in the fixation of delicate bone fragments, accompanied by facile plate adaptability. Its utility extends to cases characterized by severe comminution or small bone fragments, which are frequently encountered in patients with osteoporosis. In contrast, the use of screw fixation poses potential risks, including inadvertent fragment breakage, screw back-out, and the loosening of screws [30].

The present study has several limitations. It primarily adopted a non-comparative design, inherently susceptible to associated constraints; however, the data were meticulously extracted from an ongoing prospective database that comprehensively included all operated patients. Additionally, our investigation was confined to patients treated exclusively with the mini-plate-only fixation technique, thus lacking a comparative group to establish the method’s superiority. Second, because of the low incidence of Wagstaffe fractures, the sample size was small, and we did not collect long-term follow-up data. Consequently, future studies should include a larger cohort and an extended follow-up period. Furthermore, comparative studies among different fixation methods and biomechanical studies based on fixation materials are needed to enhance the robustness and validity of these findings. Developing metal plate configurations that can be applied more precisely to the fracture site should also be a future research focus, aiming to prevent complications. Such studies hold promise for achieving favorable outcomes in the treatment of indirect syndesmosis injuries.

## 5. Conclusions

The mini-plate fixation technique for Wagstaffe fractures demonstrated reliable fixation strength, effective fracture reduction, a low complication rate, and efficient surgical procedure duration. This method is suitable for patients with severe osteoporosis or comminuted fractures. Despite the availability of various treatment options, the mini-plate technique has emerged as a viable alternative, showing favorable outcomes for patients with Wagstaffe fractures and concurrent syndesmosis instability. However, the absence of long-term data limits the conclusions drawn from this study. Further research is necessary to confirm the technique’s effectiveness and evaluate its potential for complications over time.

## Figures and Tables

**Table 1:** Methods used for measurement.

Method	Description
Direct anterior difference	Distance between the incisura and the anterior end of the fibular orientation line, measured perpendicularly.
Direct posterior difference	Distance between the incisura and the posterior end of a line representing the orientation of the fibula, measured perpendicularly.
Fibular translation	Distance from the anterior edge of the tibial incisura to a line representing the direct anterior difference. A positive value indicated that the fibula was situated posterior to the anterior border of the incisura.
Fibular rotation	The angle between a line connecting the anterior and posterior borders of the tibial incisura and a line on the fibula representing its orientation. The angle was considered positive when the fibula was internally rotated relative to the incisura.

**Table 2:** Patient demographics.

Characteristics	Total (*n* = 17) Mean (SD) or N (%)
Age (y)	63.18 (9.38)
Sex	
Male	7 (41.2%)
Female	10 (58.8%)
Side	
Right	5 (29.4%)
Left	12 (70.6%)
Follow-up (months)	16.18 (5.30)

SD, standard deviation. Statistical analysis was performed by an expert statistician using SPSS version 29.0 software (IBM Corp., Armonk, NY, USA). Quantitative variables are expressed as mean (SD).

**Table 3:** Patient characteristics.

No.	Age (y)	Sex	Lauge-Hansen Classification	Weber Classification	Injured Side	OMAS		VAS Score	
						Pre	Post	Pre	Post
1	63	F	SER IV	B	Left	10	80	8	1
2	60	F	SER III	B	Left	25	85	7	0
3	71	F	SER IV	B	Right	5	90	8	0
4	57	M	SER II	B	Left	10	80	8	0
5	75	F	SER II	B	Left	0	80	9	2
6	58	F	PER III	C	Left	0	70	8	0
7	80	F	SER IV	B	Left	0	75	9	0
8	48	M	SER IV	B	Left	0	95	8	0
9	44	M	SER IV	B	Left	0	90	7	0
10	68	F	PER IV	C	Left	0	80	8	0
11	58	M	SER IV	B	Right	0	90	8	0
12	72	F	SER IV	B	Left	0	85	8	1
13	66	F	SER IV	B	Right	15	90	9	1
14	63	M	SER II	B	Left	0	85	8	0
15	55	F	SER IV	B	Left	25	80	9	2
16	69	M	SER IV	B	Right	15	80	8	1
17	67	M	SER IV	B	Left	15	85	8	1
Mean	63.18	NA	NA	NA	NA	5.88	83.53	8.11	0.59
SD	9.38	NA	NA	NA	NA	7.31	5.19	0.58	0.71
*p*-value						<0.001		<0.001	

No., patient number; OMAS, Olerud Molander Ankle Score; VAS, visual analog scale; Pre, preoperative; Post, postoperative; F, female; M, male; SER, supination external rotation; PER, pronation external rotation; NA, not applicable; SD, standard deviation. Note: Data are presented as the means for continuous variables and frequencies for categorical variables. The pre- and postoperative VAS and OMAS scores were compared using the Wilcoxon signed-rank test. A two-sided test with *p* < 0.05 was considered statistically significant.

**Table 4:** Results of four evaluation methods obtained by two observers.

	Observer 1			Observer 2		
Evaluation Method	Normal Side (*n* = 17)	Injured Side (*n* = 17)	*p*-Value	Normal Side (*n* = 17)	Injured Side (*n* = 17)	*p*-Value
Direct anterior difference, mm	4.57 (1.25)	4.46 (1.03)	0.587	4.49 (1.12)	4.67 (1.05)	0.698
Direct posterior difference, mm	8.42 (1.18)	8.94 (2.02)	0.300	8.32 (1.14)	9.12 (1.84)	0.549
Fibular translation, mm	1.99 (1.13)	1.67 (0.67)	0.730	1.88 (1.06)	1.73 (0.70)	0.594
Fibular rotation, degrees	11.52 (5.55)	13.35 (6.41)	0.752	11.31 (5.30)	13.43 (6.47)	0.736

Note: Data are presented as means (standard deviation) for continuous variables and as frequencies (percentages) for categorical variables. A two-sided test with *p* < 0.05 was considered statistically significant.

**Table 5:** Intra- and inter-observer reliability for the evaluation methods.

	Intraobserver Reliability		
Evaluation Method	Observer 1	Observer 2	Interobserver Reliability
Direct anterior difference, mm	0.874	0.902	0.951
Direct posterior difference, mm	0.779	0.876	0.918
Fibular translation, mm	0.862	0.854	0.935
Fibular rotation, degrees	0.948	0.940	0.975

Note: Data are presented as means for continuous variables and as frequencies for categorical variables. A two-sided test with *p* < 0.05 was considered statistically significant.

## Data Availability

Data sharing is not applicable to this article as no datasets were generated or analyzed during the current study.
